# Novel Flower-like Nickel Sulfide as an Efficient Electrocatalyst for Non-aqueous Lithium-Air Batteries

**DOI:** 10.1038/srep18199

**Published:** 2015-12-14

**Authors:** Zhong Ma, Xianxia Yuan, Zhenlin Zhang, Delong Mei, Lin Li, Zi-Feng Ma, Lei Zhang, Jun Yang, Jiujun Zhang

**Affiliations:** 1 Department of Chemical Engineering, Shanghai Jiao Tong University, Shanghai, 200240, China; 2 NRC Energy, Mining & Environment Portfolio, National Research Council Canada, Vancouver, BC V6T 1W5, Canada

## Abstract

In this paper, metal sulfide materials have been explored for the first time as a new choice of bifunctional cathode electrocatalyst materials for non-aqueous lithium-air batteries (LABs). Nickel sulfides with two different morphologies of flower-like (f-NiS) and rod-like (r-NiS) are successfully synthesized using a hydrothermal method with and without the assistance of cetyltrimethyl ammonium bromide. As LAB cathode catalysts, both f-NiS and r-NiS demonstrate excellent catalytic activities towards the formation and decomposition of Li_2_O_2_, resulting in improved specific capacity, reduced overpotentials and enhanced cycling performance when compared to those of pure Super P based electrode. Moreover, the morphology of NiS materials can greatly affect LAB performance. Particularly, the f-NiS is more favorable than r-NiS in terms of their application in LABs. When compared to both r-NiS and pure super P materials as LAB cathode materials, this f-NiS catalyst material can give the highest capacity of 6733 mA h g^−1^ and the lowest charge voltage of 4.24 V at the current density of 75 mA g^−1^ and also exhibit an quite stable cycling performance.

With the fast emerging of electric vehicles (EVs), the urgent demand for improving the energy and power densities of electrochemical energy storage and conversion technologies such as batteries, fuel cells, and supercapacirors has become more and more strong in recent years^1^,^2^. Lithium-air batteries (LABs), owing to their expected high practical specific energy densities from 1000 to 2000 Wh kg^−1^ which could even match with 1700 Wh kg^−1^ of gasoline energy system^3^,^4^,^5^, are considered to be the most promising of known rechargeable battery technologies to provide enough energy storage capability for EVs to drive more than 500 miles (per charge)^3^,^4^,^5^. However, LABs are still at an early stage of development currently due to their unrealized specific capacity/energy density, rate discharge-ability, capacity sustainability as well as round-trip efficiency^4^,^6^. It has been identified that among various issues affecting LAB performance, low performing air cathode, especially the cathode catalyst, is the dominating challenge^7^,^8^,^9^,^10^ although Li anode^11^, operating atmosphere^12^, binder^13^, cathode process^14^, and solvents^15^ as well as lithium salts^16^ in the electrolyte could also make contributions to the overall battery performance.

Regarding the catalytic electrode materials, non-noble transition metal oxides (TMOs) have been widely used in many electrochemical devices in recent decades, including fuel cells^17^, Li-ion batteries^18^, Li-air batteries and industry catalytic systems^19^. For example, since the early introduction of MnO_2_ into LABs in 2006^20^, a huge number of TMOs, such as Co_3_O_4_^21^, Fe_2_O_3_^22^ and NiO^23^,^24^, NiCo_2_O_4_^25^ and perovskite oxide^8^,^26^, have been explored as cathode catalyst materials for LABs due to their good catalytic performance towards cathode reactions of both oxygen reduction reaction (ORR) and oxygen evolution reaction (OER). Considering the similar electron structures of oxygen and sulfur, transition metal sulfides (TMSs), as another group of transition metal compounds, have also been expected to have similar roles in electrochemical devices^27^,^28^,^29^. However, TMSs have not been reported to work as the bifunctional cathode catalysts in LABs to date.

In this work, nickel sulfide (NiS), as a member of TMSs, is employed as the bifunctional cathode catalyst for non-aqueous LABs for the first time. To study the possible effects of morphology/structure of catalyst on its performance, both flower-like nickel sulfide (f-NiS) and rod-like nickel sulfide (r-NiS) are successfully synthesized by a hydrothermal method with and without the assistance of cetyltrimethyl ammonium bromide (CTAB), respectively. Compared to the pure Super P carbon based LAB cathode, NiS as a catalyst material shows significantly improved specific capacity, reduced discharge/charge overpotentials, and enhanced cycling performance. Moreover, the flower-like architecture of NiS is more favorable than the rod-like one in terms of performance enhancement.

## Results

### Synthesis and characterization of NiS catalysts

**Figure 1:**
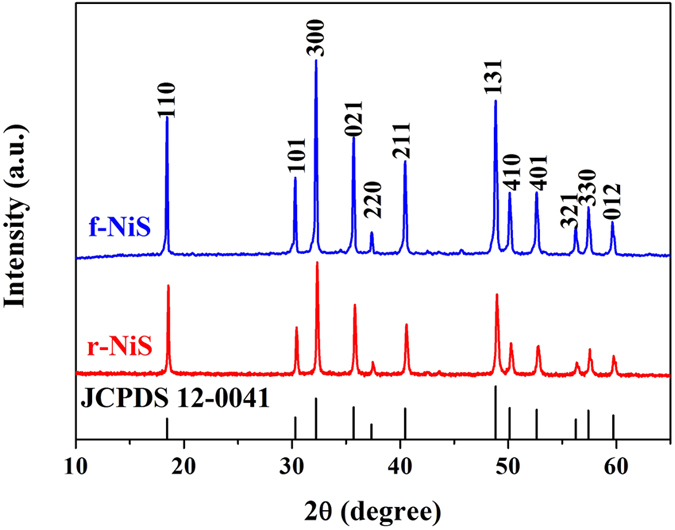
XRD patterns of as-prepared f-NiS and r-NiS catalysts.

**Figure 2:**
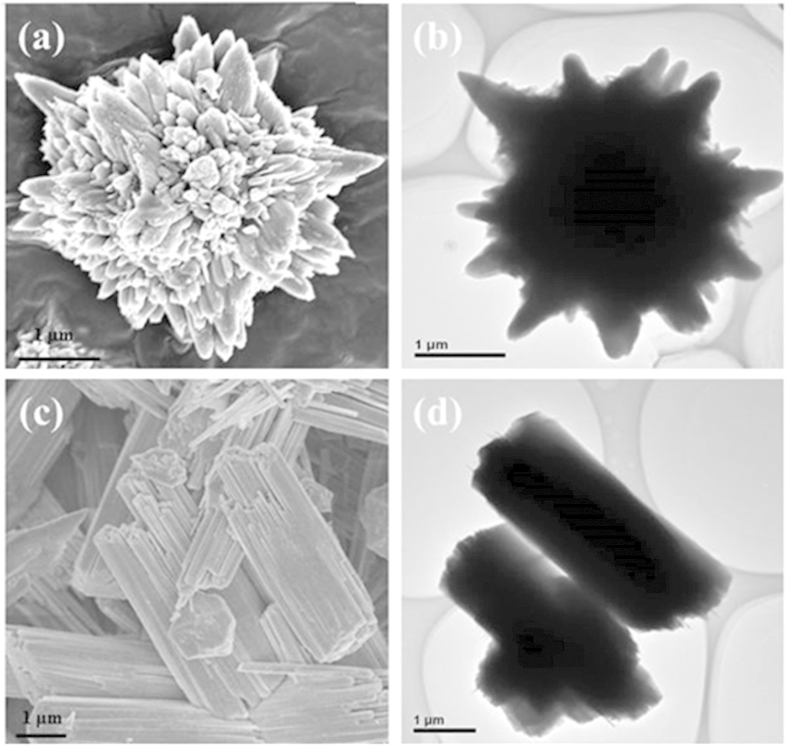
SEM (a) and TEM (b) images of f-NiS; SEM (c) and TEM (d) images of r-NiS.

The f-NiS and r-NiS were prepared by a hydrothermal method with and without the assistant of cetyltrimethyl ammonium bromide (CTAB), respectively, using Ni(NO_3_)_2_·6H_2_O and NaSCN as the precursors at 220 °C for 24 hours^30^. Figure 1 presents the XRD patterns of the as-prepared f-NiS and r-NiS catalysts. Both of them have the same crystal structures and all of the characteristic peaks agree well with the standard pattern of rhombohedral crystalline NiS (JCPDS 12-0041). Furthermore, none impurities could be seen from the XRD patterns. Both the field emission scanning electron microscopy (FESEM) and transmission electron microscope (TEM) were also performed to observe the morphologies/microstructures of the as-prepared f-NiS and r-NiS materials. As shown in Fig. 2a and Figure S1, the as-prepared f-NiS catalyst is consisted of a uniform and discrete flower-like structure made up with multilayered and highly ordered texture, and the flower-like particles are several micrometers in size and composed of both nanopetals and nanorods. The EDX results displayed in Figure S2 (where the signals of C and O are coming from the conductive carbon tape, and that of Pt is from the platinum sputtering for conductivity improvement during the preparation of SEM samples) indicate the elements of Ni and S with a molar ratio of about 1:1 (Ni:S), confirming again the pure NiS in the as-prepared sample as discussed above with the XRD data. The TEM image as Fig. 2b indicates a solid architecture of the f-NiS. The growth of such a f-NiS could be explained in terms of the diffusion-limited aggregation (DLA) process and the cage effect^30^, which should be induced by the addition of CTAB. This CTAB could separate the solution system into plenteous cages that might contain limited amounts of Ni^2+^ and [Ni(SCN)_x_]^2−x^ (0 ≤ x ≤ 4). The S^2−^ released from [Ni(SCN)_x_]^2−x^ could then collide with uncombined Ni^2+^ to form NiS with petal and/or rod particles. Figure 2c, Figure S3 and Fig. 2d depict the SEM and TEM images of the as-prepared r-NiS, showing a solid rod-like morphology/structure with a size of ~1.5 μm. It is believed that its formation is through a flake-cracking mechanism^31^, where Ni^2+^ and [Ni(SCN)_x_]^2−x^ (0 ≤ x ≤ 4) react to produce flake-like NiS, and the flakes then crack and form the nanorods with smaller diameters with increasing reaction time.

### Electrochemical performance of NiS catalysts in LABs

**Figure 3:**
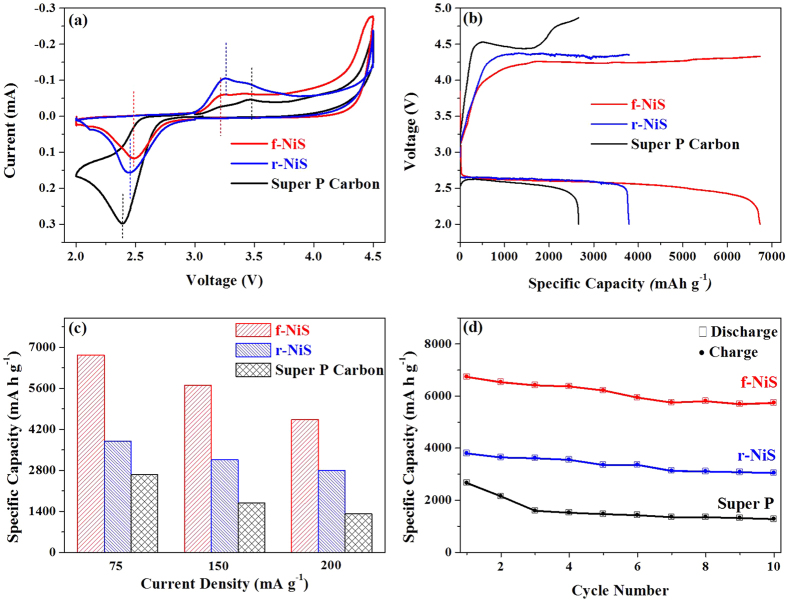
(**a**) Cyclic voltammonograms (CVs) of NiS and Super P based cathodes in oxygen saturated 1 M LiTFSI/TEGDME electrolyte at 0.5 mV s^−1^; (**b**) Discharge-charge profiles of LABs with NiS and Super P based cathodes at 75 mA g^−1^; (**c**) Specific capacities of NiS and Super P based cathodes at various current densities (75, 150 and 200 mA g^−1^); (**d**) Discharge/charge capacities versus cycle number for NiS and Super P based cathodes at 75 mA g^−1^ (**d**).

Two as-prepared NiS materials (f-NiS and r-NiS) were examined as cathode catalysts for non-aqueous LABs. The cathodes were prepared by mixing catalyst, carbon material (Super P) and binder (PVDF) with a weight ratio of 3:6:1. For comparison, a pure carbon based cathode was also prepared with only Super P and binder with a ratio of 9:1. Figure 3a shows the cyclic voltammograms (CV curves) of f-NiS, r-NiS and Super P based cathodes in an oxygen saturated 1 M LiTFSI/TEGDME electrolyte in the voltage range of 2.0–4.5 V at 0.5 mV s^−1^. It can be seen that both of the f-NiS and r-NiS based electrodes demonstrate higher ORR and OER kinetics (more positive ORR peak potentials and less positive OER peak potentials) than the Super P electrode. A comparison between f-NiS and r-NiS based cathodes indicates that, f-NiS has a slightly better ORR activity and considerably higher OER activity than that of the r-NiS, suggesting that f-NiS based LAB might exhibit a best performance with lowest charge potential. This can be confirmed by the discharge-charge profiles at a current density of 75 mA g^−1^, as displayed in Fig. 3b, where the f-NiS based LAB delivers a largest discharge capacity of 6733 mA h g^−1^, which is significantly higher than that of 3794 and 2661 mA h g^−1^ for r-NiS and Super P based batteries, respectively. The f-NiS based LAB also displays a lowest charge potential of 4.24 V, which is 130 mV and 300 mV lower, respectively, than those of the r-NiS and Super P based LABs. It is also noticed that both of the NiS based LABs have similar discharge potentials which are slightly higher than that of Super P based LAB. When the current density is increased to 150 and 200 mA g^−1^, respectively, the f-NiS based LAB retains capacities of 5704 and 4532 mA h g^−1^ (Fig. 3c), which are almost 2 times higher than that of the Super P based one. In addition, the capacities of the f-NiS based LAB at several elevated current densities are considerably larger than those of the r-NiS based one. These results indicate that both NiS based electrodes have enhanced specific capacities, reduced overpotentials and improved rate capability when compared to those of the Super P electrode, and the f-NiS is more favorable than r-NiS as the cathode catalyst material.

### Cycling performance of LABs with NiS catalysts

**Figure 4:**
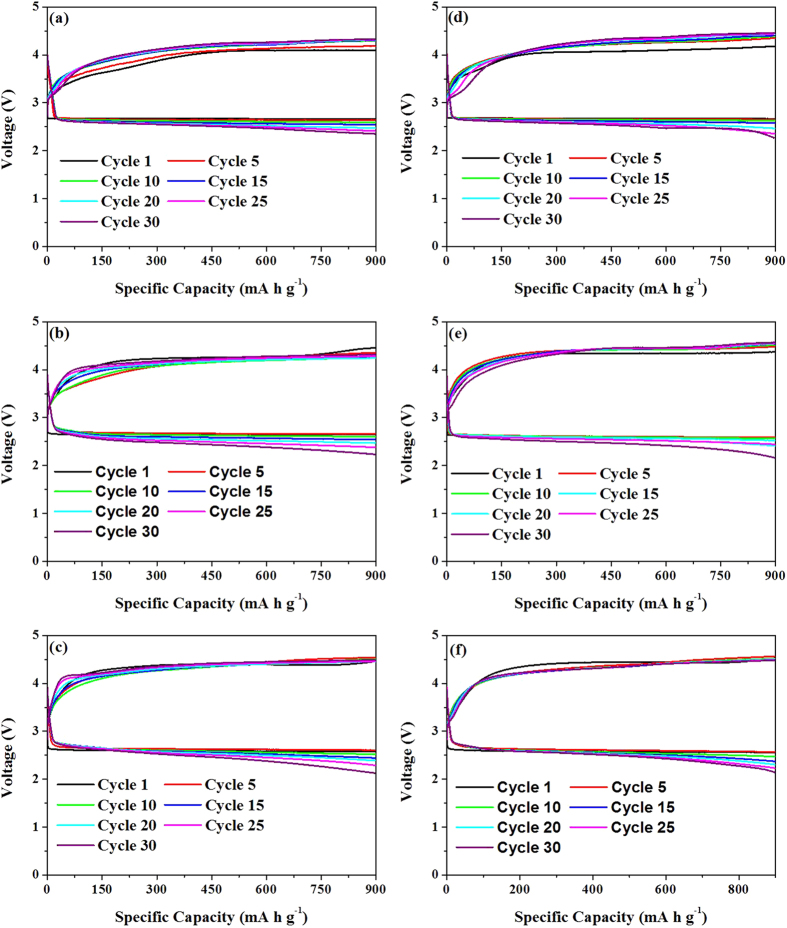
Cycling performance of f-NiS (**a–c**) and r-NiS (**d–f**) based electrodes with controlled capacity of 900 mA h g^−1^ at various current densities (75 (**a,d**),150 (**b,e**) and 200 (**c,f**) mA g^−1^.

The discharge-charge cycling performance of LABs with NiS catalysts as well as Super P carbon was investigated in a potential range from 2.0 to 4.5 V at a current density of 75 mA g^−1^. As shown in Fig. 3d, the f-NiS and r-NiS based LABs exhibit discharge capacities of 5737 and 3043 mA h g^−1^, respectively, at the 10th cycle, while only 1277 mA h g^−1^ could be achieved for the pure Super P based one. The capacity retention of f-NiS based LAB is 85%, which is higher than that of 80% and 48% for r-NiS and Super P based ones, respectively. However, the LAB with both f-NiS and r-NiS catalysts still suffer a certain extent of capacity fading during the full capacity discharge-charge cycling. In literature, in evaluating the cathode catalysts, the cycling performance is usually tested by setting a constant capacity or cut-off voltage^32^. In the present work, the cycling performance of both f-NiS and r-NiS based LABs was examined by controlling a discharge depth of 900 mA h g^−1^ at current densities of 75, 150 and 200 mA g^−1^, respectively (Fig. 4 and Figure S4). For comparison, the same experiment was also conducted on a pure Super P based LAB (Figure S5). It can be seen that the LABs with both f-NiS and r-NiS catalyst materials can sustain a specific capacity of 900 mA h g^−1^ for over 30 cycles without fading to below 2.0 V regardless of the current density. However, the one with Super P cathode can only run about 24, 17 and 12 cycles at 75, 150 and 200 mA g^−1^, respectively, before the discharge terminal voltage falls down to below 2.0 V. As for the two NiS based LABs, the one with f-NiS can sustain a higher discharge terminal voltage, lower charge terminal voltage and slower fading after 30 cycles. For example, the f-NiS based LAB with initial terminal discharge and charge voltages of 2.66 and 4.09 V can retain at 2.35 and 4.33 V, respectively, after 30 cycles at 75 mA g^−1^. While the r-NiS based one with its initial voltages of 2.66 and 4.18 V presents at 2.27 and 4.46 V, respectively, after 30 cycles. When the current density is increased to 150 mA g^−1^, the terminal discharge and charge voltages of f-NiS based LAB can still attain at 2.23 and 4.33 V, respectively, after 30 cycles, compared to that of 2.14 and 4.57 V for the r-NiS based one. Based on the results discussed above, one can conclude that both f-NiS and r-NiS can achieve enhanced cycling performance when compared to the pure Super P based LAB, and the flower-like one is more favorable than the rod-like one when used as the LAB cathode catalyst material.

### Characterization and morphology analysis of cathode products in NiS-based electrodes

**Figure 5:**
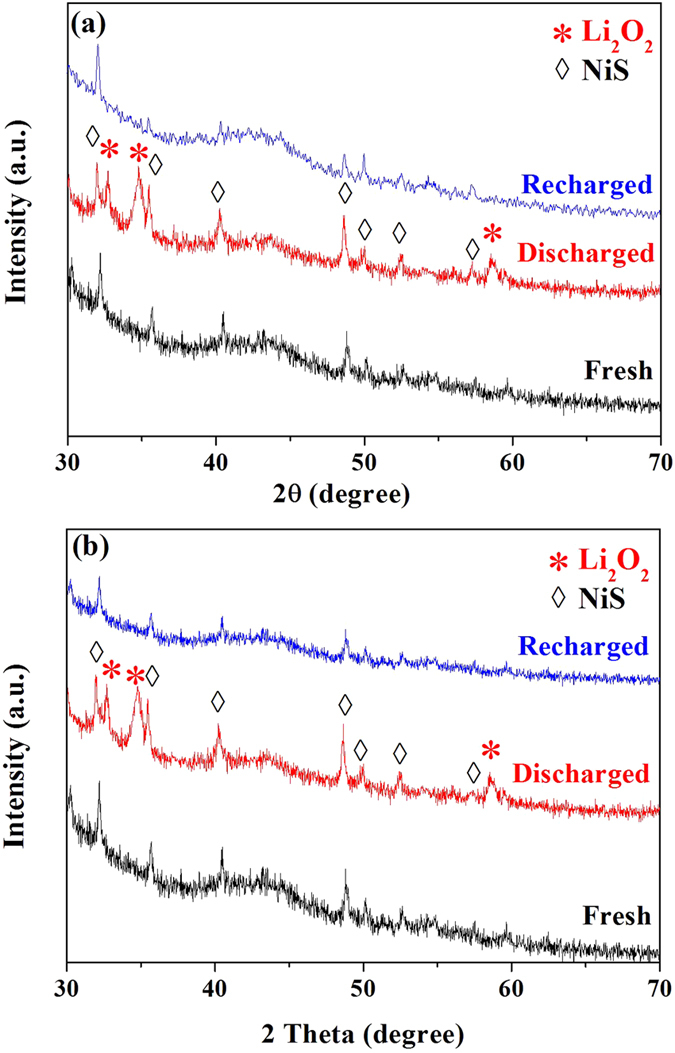
XRD patterns of fresh, discharged and recharged cathodes with f-NiS (a) and r-NiS (b) as catalysts.

**Figure 6:**
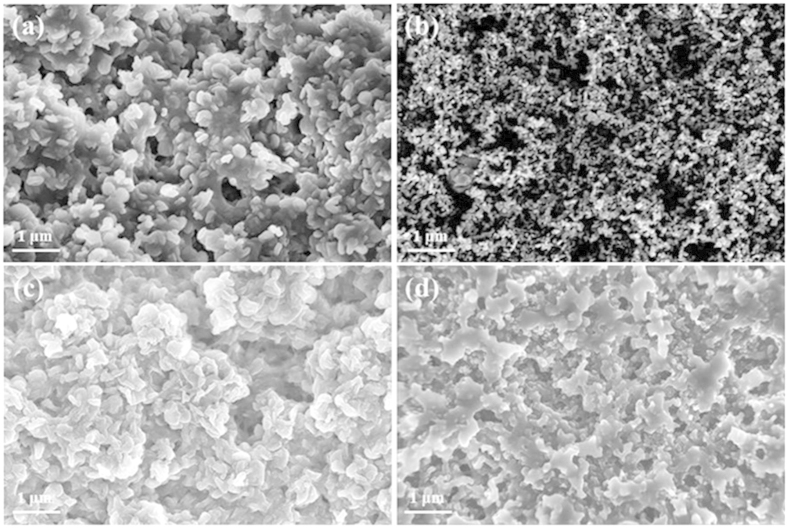
SEM images of the f-NiS (**a,b**) and r-NiS (**c,d**) based electrodes at various states: discharged (**a,c**) and recharged (**b,d**).

The category and morphology of the cathode products that can generally affected by the catalysts used in LABs are important aspects influencing the battery performance^33^,^34^,^35^. Thus, XRD and SEM measurements were conducted in this work to characterize the phase structure and morphology of the cathode products in the electrodes at various states for obtaining a further insight into the superior performance of the NiS catalyst materials especially the flower-like NiS. Figure 5 displays the acquired XRD patterns for both f-NiS and r-NiS based cathodes. In all of the studied electrodes, the peaks at 32.2°, 35.7°, 40.5°, 48.8°, 50.1°, 52.6° and 57.4°, which correspond to rhombohedral crystalline NiS as discussed above with Fig. 1, could be clearly observed, indicating the stable phase structure of NiS during the LAB operation. In the discharged cathodes with both NiS materials, three new strong peaks centered at 32.9°, 35° and 58.6°, respectively, can be clearly observed, implying the formation of Li_2_O_2_ during the discharge process. When the electrodes are recharged back, however, all the peaks corresponding to Li_2_O_2_ disappear and the XRD patterns become almost the same as that of the fresh electrode, indicating a complete decomposition of Li_2_O_2_ in the NiS-based cathodes. In the pure Super P based electrodes (Figure S6), however, there are only two weak peaks at 32.9° and 35° that could be assigned to Li_2_O_2_ in the discharged electrode, indicating a poor crystallization of Li_2_O_2_ during the discharge process. These peaks, especially the one at 35°, could still be observed in the recharged electrode, implying an incomplete decomposition of Li_2_O_2_ during the charge process. The morphologies of f-NiS, r-NiS and Super P based electrodes at various states were investigated with SEM. As shown in Figure S7, the flower-like morphology of f-NiS and the rod-like morphology of r-NiS are maintained well in the fresh electrode, indicating that the fabrication process doesn’t destroy the special morphology of f-NiS and r-NiS. Figure 6 shows the morphologies of the discharged and recharged cathodes with both NiS materials, a large difference can be observed. The discharge products exist as some separated toroid-like particles and disperse uniformly on the surface of f-NiS based cathode, while the flake architectural particles overlapping each other are formed on the surface of r-NiS based electrode. The particle sizes of the discharge products on both cathodes are evidently different. It is about 200–300 nm on the f-NiS based electrode, which is obviously smaller than that of 300–400 nm on the r-NiS based electrode. In the Super P based cathode (Figure S8), some toroid-like discharge products connect into a thin film on the surface. Moreover, the discharge products on both cathodes with NiS catalyst materials have a hierarchical and porous structure, while that on the Super P based electrode is densely accumulated. At the recharged state, almost all of the discharge products disappear when f-NiS is used as the catalyst, and a small amount of discharge products could still be observed on the cathode employing r-NiS catalyst. In the pure Super P based electrode, however, many particles of discharge products can be clearly seen, indicating an incomplete decomposition of the discharge products, Li_2_O_2_, during the charge process. This agrees well with the observation from the XRD patterns discussed above. Thus, it could be figured out that NiS catalysts possess excellent capability to facilitate the formation and decomposition of Li_2_O_2_ as discharge products on the cathode of LABs.

## Discussion

The results displayed above have demonstrated that NiS catalysts can not only improve the specific capacity of LABs, but also exhibit both superior ORR and OER activities towards the formation and decomposition of Li_2_O_2_, which lead to decreased overpotentials, increased high-rate capability and enhanced cycling performance. Furthermore, the morphology of NiS has a great influence on LAB performance, and the flower-like NiS is more capable than the rod-like one in enhancing the battery performance. The excellent activity of NiS catalysts, especially the f-NiS, and their superior performance in LABs may be attributed to the following four main factors: (1) the intrinsic properties of NiS, as a member of TMSs (which are similar to TMOs), make it capable of enhancing the kinetics of Li_2_O_2_ formation/decomposition^36^, which can not only enhance the specific capacity by raising pore structure utilization in the electrode and increase the round trip efficiency, but also extend the cycling life by entirely releasing the pore structure of electrode through complete decomposition of discharge products; (2) the hierarchical and porous structure of the discharge products in the cathodes with NiS could benefit the durable and uniform diffusion of oxygen, resulting in a sustainment of discharge process for achieving higher specific capacity^37^,^38^; (3) the flower-like architecture (3D structure) could provide more space for the storage of discharge products and more channels for oxygen diffusion, which might be the reason for its highest specific capacity^39^,^40^; and (4) the morphology of discharge products could be tailored by cathode catalyst^41^ leading to different battery performance. The uniformly dispersed toroid-like particles with smaller particle sizes on f-NiS cathode could be easily decomposed compared to the larger overlapped flakes on r-NiS electrode, thereby leading to lower charge potentials^42^. These lower potentials could avoid the occurrence of side reactions and prevent the pore structure blocking from irreversible products, then result in enhanced cycling performance.

## Conclusion

In summary, NiS catalyst materials with flower-like and rod-like architectures are successfully synthesized using a hydrothermal method, and employed for the first time as cathode catalysts for LABs. Compared to Super P carbon materials, NiS catalysts give a much better performance in terms of the specific capacity, discharge/charge overpotentials, rate dischargeability and cycling performance. Moreover, tuning the structure of NiS could enhance the performance of their associated LABs through tailoring the channels for oxygen diffusion and the space for discharge products storage, as well as the morphology/structure of discharge products. Particularly, the LABs assembled with f-NiS catalyst material can give a higher specific capacity, lower charge overpotential, better high-rate dischargeability and more stable cycling performance than r-NiS based batteries. This work suggests that f-NiS is a promising candidate for LAB cathode catalyst material, and in general, TMSs might be a novel family of high efficient cathode catalysts for non-aqueous rechargeable LABs.

## Methods

### Synthesis of flower-like and rod-like Nickel Sulfides

The synthesis of flower-like nickel sulfide (f-NiS) was referred to a previous report^30^, the typical process was as follows: 1.7 mmol Ni(NO_3_)_2_·6H_2_O, 1.8 mmol NaSCN and 2.58 mmol cetyltrimethyl ammonium bromide (CTAB) were dissolved in 80 ml ultrapure water to form a solution. The solution was then transferred into a Teflon-lined autoclave (150 ml in capability) sealed with a stainless steel jar. When the solution became clear and bright green, the autoclave was heated to 220 °C and maintained at this temperature for 24 hours. After cooling down to room temperature, the flower-like nickel sulfide (f-NiS) was collected by centrifugation and washed thoroughly with distilled water and ethanol alternate for several times followed by a drying step in vacuum at 80 °C overnight. To synthesize rod-like nickel sulfide (r-NiS), the same procedure was employed but without using CTAB.

### Structural and morphology characterizations

The phase components and structure of the as-prepared NiS materials and their air cathodes at various states were characterized by powder X-ray diffraction (XRD) using a Bruker D8 advance diffractometer. The morphologies were analyzed with FEI Nova SEM 230 ultra-high resolution field emission scanning electron microscope (FESEM) equipped with Oxford INCA X-Max 80 and JEOL JEM-2010HT transmission electron microscopy (TEM).

### Fabrication of Li-air batteries

The air cathodes were prepared with a coating method as reported in our previous work^14^ employing carbon paper (HCP010; thickness: 0.1 mm; density: 0.78 g m^−3^; Shanghai Hesen Electrical Co., Ltd. China) as the substrate. NiS catalysts, Super P carbon (SCM Industrial Chemical Co., Ltd.) and polyvinylidene fluoride (PVDF) with the weight ratio of 3:6:1 were dispersed in N-methyl-2-pyrrolidone (NMP) to make a slurry which was coated onto the carbon paper substrate. For comparison, the pure carbon cathodes without catalysts were fabricated with the slurry made of 90 wt.% Super P carbon and 10 wt.% PVDF using the same procedure. An as-prepared air electrode, a lithium metal anode, a glass fiber separator, and an electrolyte of 1 M LiTFSI (lithium bis-(trifluoromethanesulfonyl)-imide) in TEGDME (tetrathylene glycol dimethyl ether) were used together to assemble a LAB with a self-modified Swagelok-type cell^14^ in an argon-filled glove box with oxygen and water contents less than 0.1 ppm.

### Electrochemical measurements

The cyclic voltammograms (CV curves) for f-NiS, r-NiS and pure Super P based cathodes were recorded using a CHI 750a electrochemical potentiostat/galvanostat within the voltage range of 2.0–4.5 V (*vs*. Li metal) under O_2_ atmosphere with a potential scanning rate of 0.5 mV s^−1^ at room temperature. The electrochemical performance of the batteries was measured in a 1.0 atm O_2_ atmosphere using a LAND CT2001A battery testing system at room temperature. Before the galvanostatic discharge/charge measurements, the batteries were placed in a flowing pure oxygen for 1 hour and then transferred into an oxygen filled glass container for 6 hours. The specific capacity and current density were calculated based on the amount of carbon material in the cathode.

## Additional Information

**How to cite this article**: Ma, Z. *et al.* Novel Flower-like Nickel Sulfide as an Efficient Electrocatalyst for Non-aqueous Lithium-Air Batteries. *Sci. Rep.* **5**, 18199; doi: 10.1038/srep18199 (2015).

## Supplementary Material

Supplementary Information
