# Supplementary material for: Novel Flower-like Nickel Sulfide as an Efficient Electrocatalyst for Non-aqueous Lithium-Air Batteries
Source: Sci Rep. 2015 Dec 14;5:18199. doi: 10.1038/srep18199 (PMC4677382; doi:10.1038/srep18199)
Supplement: Supplementary Information [file srep18199-s1.pdf]

Supplementary Information

Novel Flower-like Nickel Sulfide as an Efficient Electrocatalyst for Non-aqueous Lithium-Air Batteries

Zhong Ma[1], Xianxia Yuan[1,*], Zhenlin Zhang[1], Delong Mei[1], Lin Li[1], Zi-Feng Ma[1], Lei Zhang[2], Jun Yang[1], Jiujun Zhang[2]

[1] Department of Chemical Engineering, Shanghai Jiao Tong University, Shanghai, 200240, China

[2] NRC Energy, Mining & Environment Portfolio, National Research Council Canada, Vancouver, BC V6T 1W5, Canada

[*]Corresponding authors: yuanxx@sjtu.edu.cn (X. Yuan)

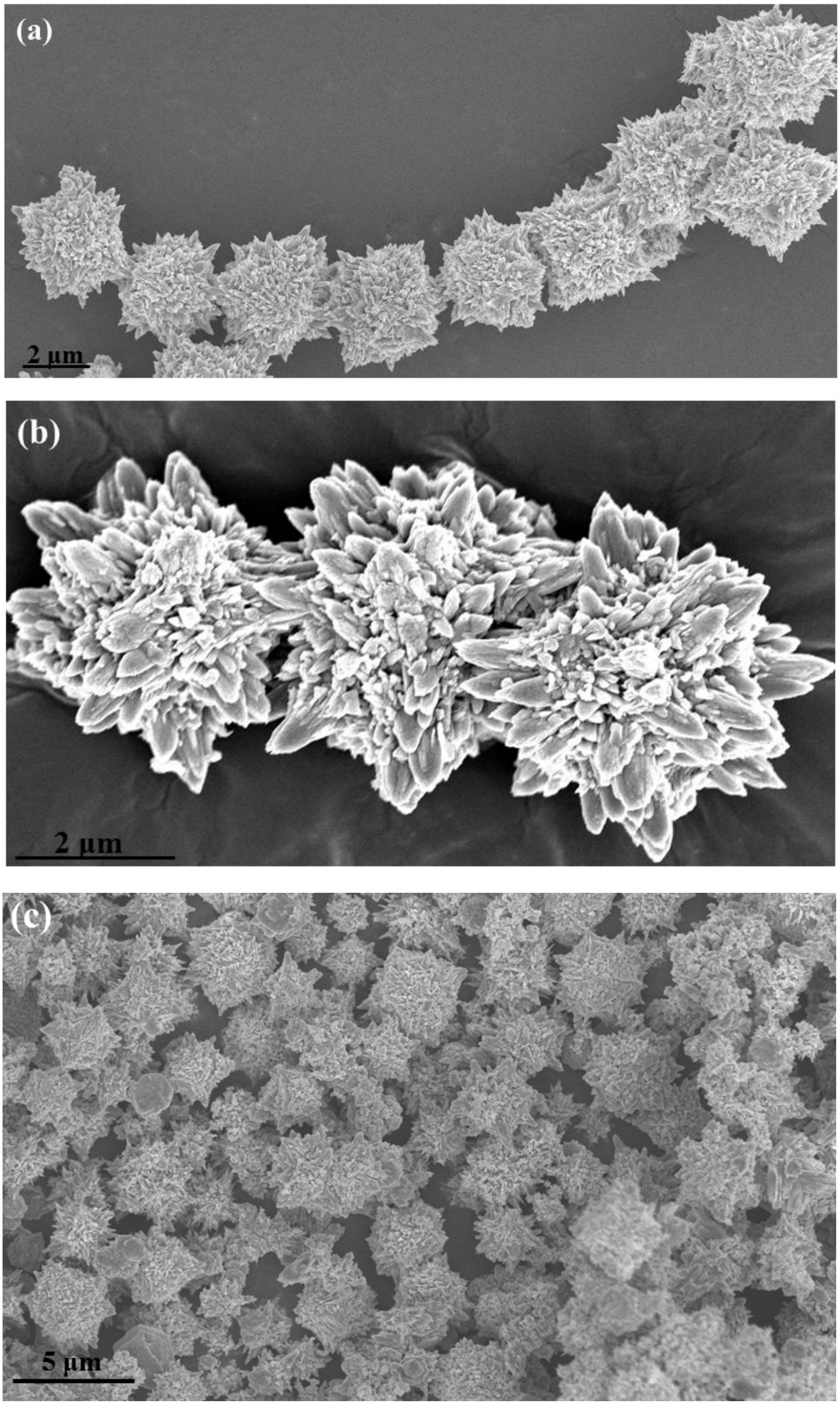

Figure S1 SEM images of the as-prepared f-NiS catalysts

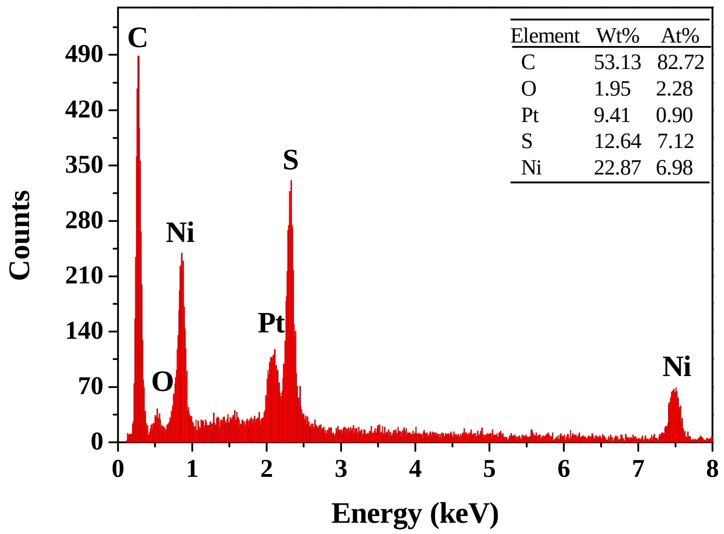

Figure S2 EDX of the as-prepared f-NiS catalyst

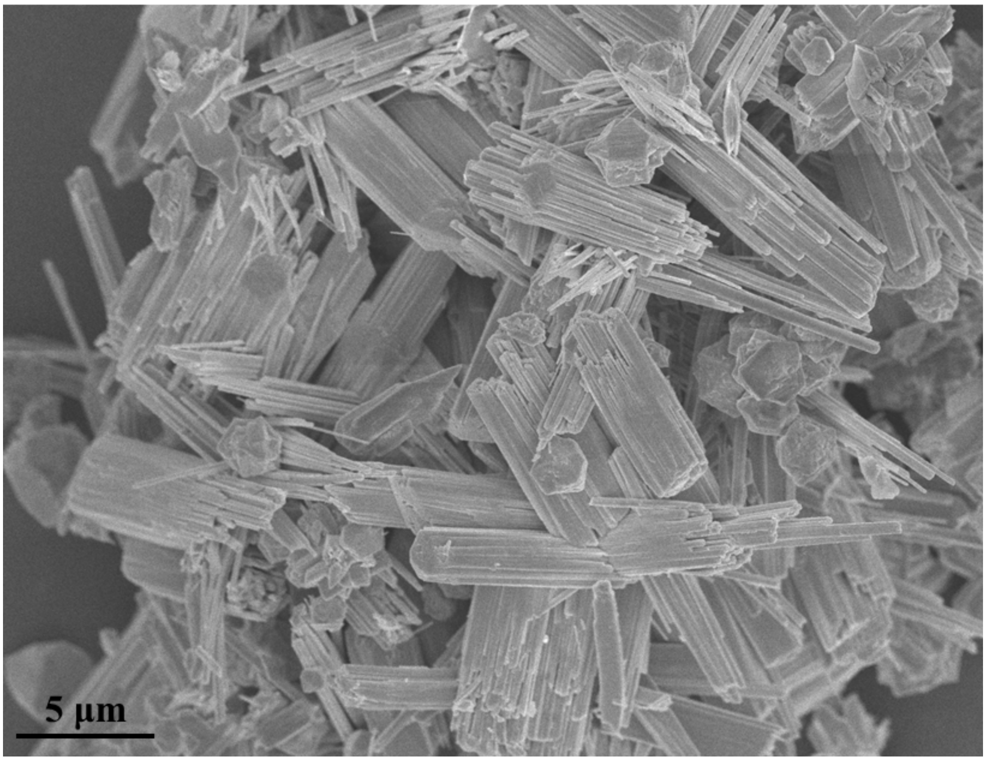

Figure S3 SEM images of the as-prepared r-NiS catalysts

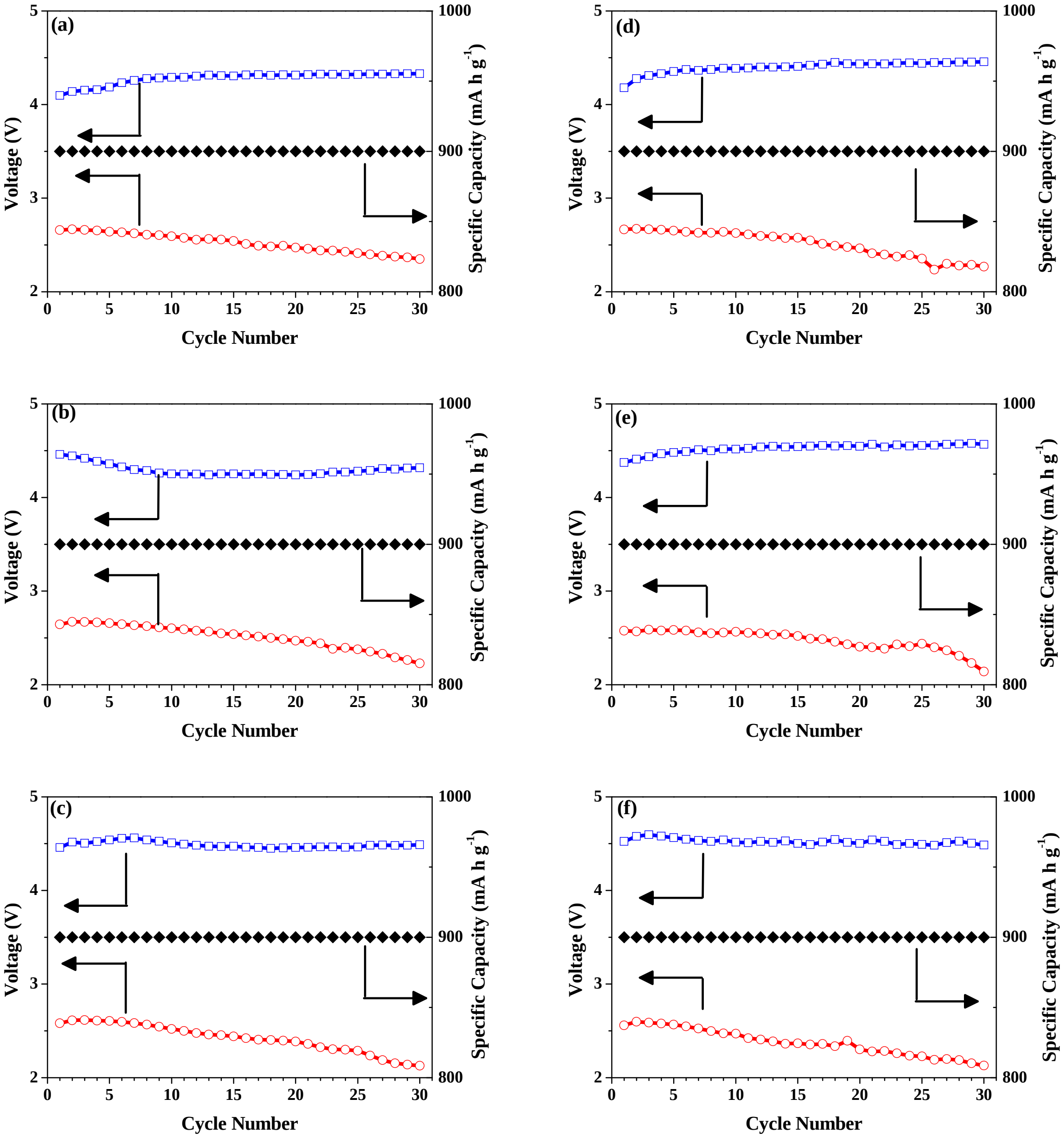

Figure S4 Terminal voltages versus cycle number for f-NiS (a, b, c) and r-NiS (d, e, f) based electrodes with a controlled capacity of 900 mA h g^{-1} at various current densities (75 (a, d),150 (b, e) and 200 (c, f) mA g^{-1})

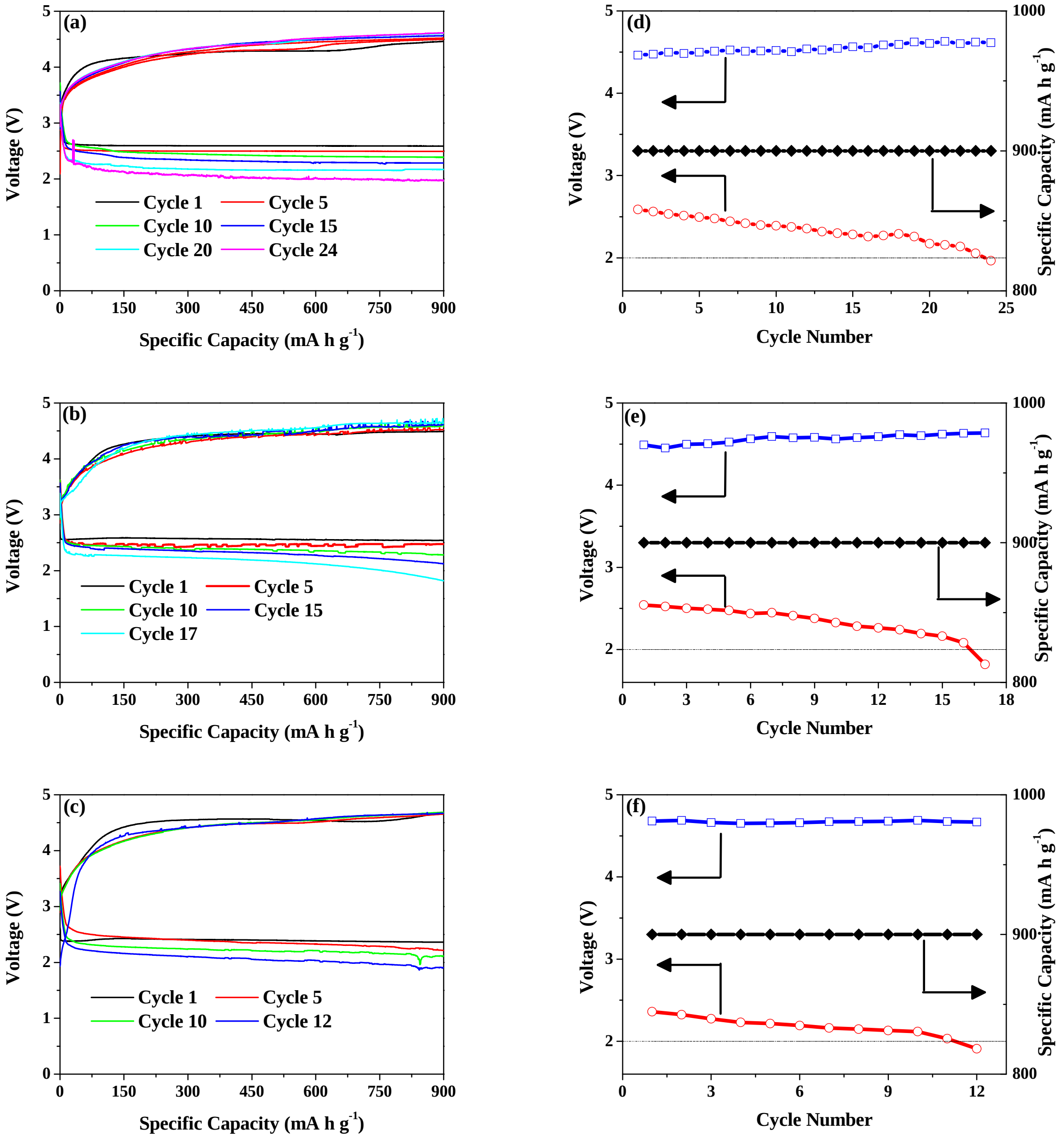

Figure S5 Discharge-charge profiles and terminal voltages of LABs with pure Super P cathode versus cycle number at various current densities (75 (a, d), 150 (b, e) and 200 (c, f) mA g^{-1})

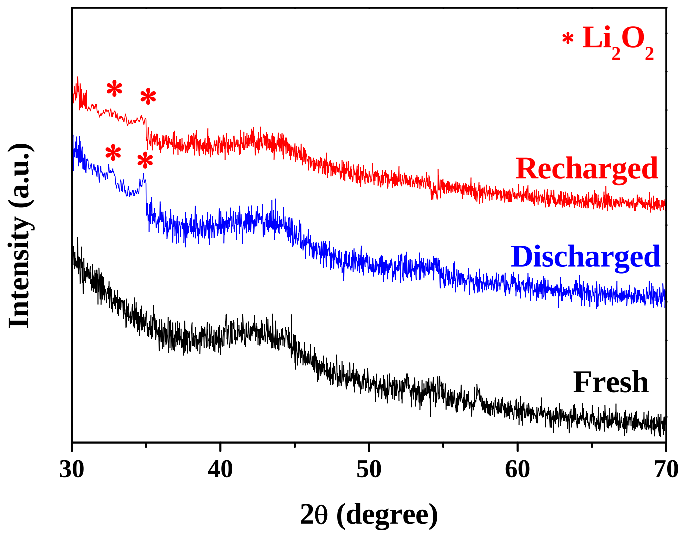

Figure S6 XRD patterns of fresh, discharged and recharged pure Super P based cathodes

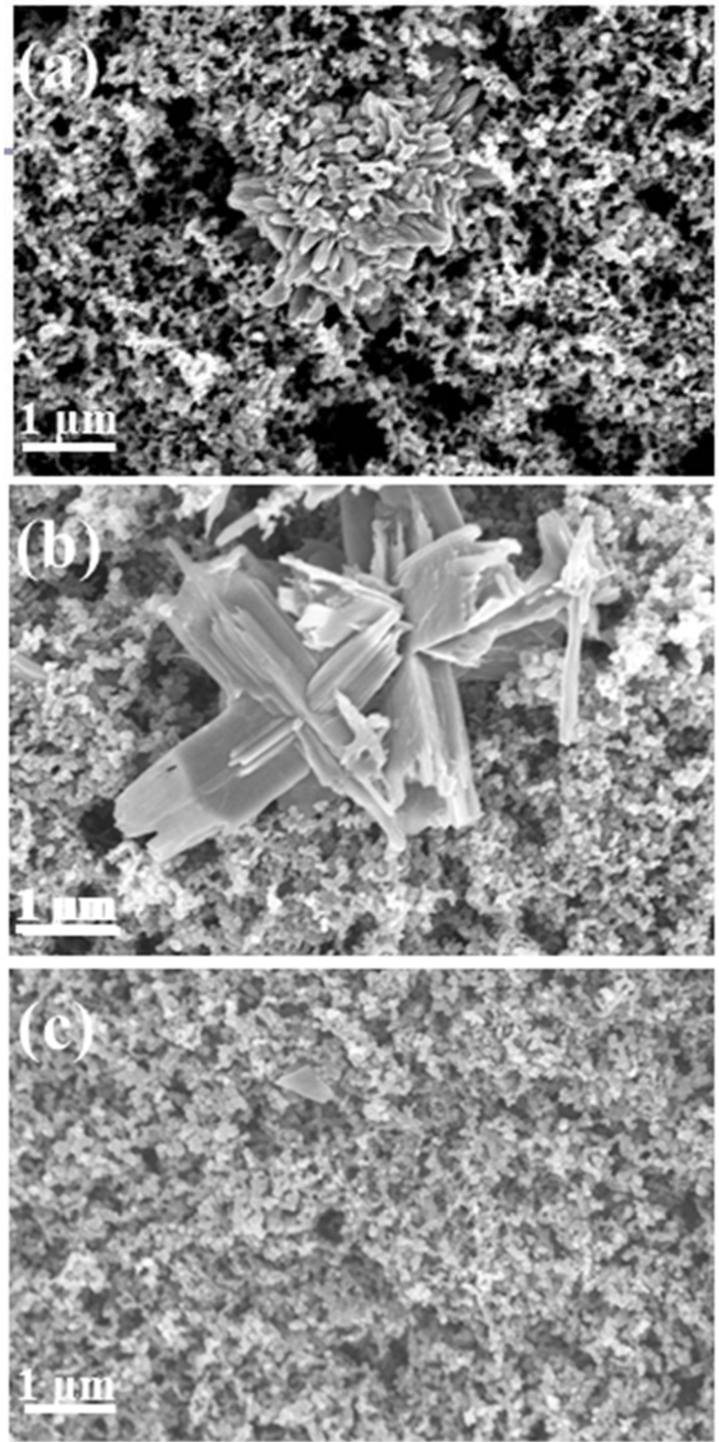

Figure S7 SEM images of fresh electrodes: f-NiS (a), r-NiS (b) and Super P (c)

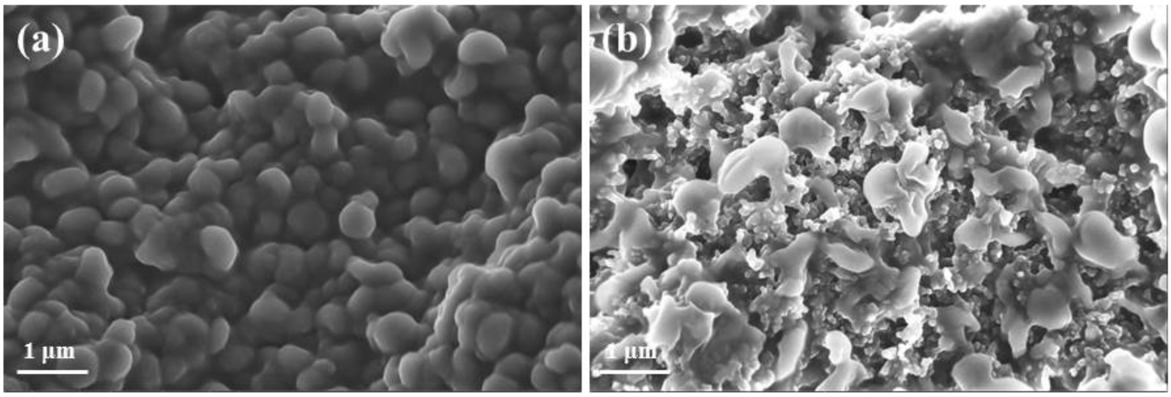

Figure S8 SEM images of pure Super P based cathodes: discharged (a) and recharged (b).
